# Comparison of Clinical Characteristics Between Febrile and Afebrile Seizures Associated With Acute Gastroenteritis in Childhood

**DOI:** 10.3389/fped.2020.00167

**Published:** 2020-04-16

**Authors:** Yan-Zhang Wu, Yao-Hua Liu, Chien-Ming Tseng, Yung-Hao Tseng, Tai-Heng Chen

**Affiliations:** ^1^Division of Pediatric Emergency, Department of Pediatrics, Kaohsiung Medical University Hospital, Kaohsiung Medical University, Kaohsiung, Taiwan; ^2^Department of Emergency, Kaohsiung Municipal Siaogang Hospital, Kaohsiung Medical University, Kaohsiung, Taiwan; ^3^Ph.D. Program in Translational Medicine, Graduate Institute of Clinical Medicine, Kaohsiung Medical University and Academia Sinica, Kaohsiung, Taiwan; ^4^Faculty of Medicine, College of Medicine, Kaohsiung Medical University, Kaohsiung, Taiwan

**Keywords:** seizures, acute gastroenteritis, fever, afebrile, comparison, clinical features

## Abstract

**Background:** Acute gastroenteritis (AGE) accompanied by seizures is not a rare scenario in childhood. We investigated the clinical features of children with febrile or afebrile seizures during AGE and aimed to identify the impact of fever in this situation-related seizure.

**Methods:** We retrospectively reviewed the medical charts of children admitted due to seizures associated with mild AGE between January 2008 and December 2017. These consecutive patients were divided into two groups: an “afebrile group” whose diagnosis was compatible with “benign convulsion with mild gastroenteritis (CwG)” and a “febrile group” who had a fever within 24 h of the onset of an AGE-related seizure. We compared the two groups' clinical and laboratory characteristics, electroencephalograms (EEG), neuroimaging, and outcomes.

**Results:** Of the children suffering from AGE and seizures, 41 were afebrile and 30 were febrile, with a mean age of 32.2 ± 27.6 months. The gender, seizure semiology, frequency, duration of seizures, the time interval between AGE symptoms onset and first seizure, and levels of serum sodium, and hepatic enzymes were significantly different between the two groups. The most frequently identified enteropathogen was rotavirus (33%), especially in the male and febrile subjects. Afebrile patients had more EEG abnormalities initially, but all returned to normal later. All cases had an uneventful outcome. Of note, seizure clusters (≥2 episodes) occurred more frequently in the afebrile patients who had a duration of AGE symptoms lasting 2 days or more, or white blood cell counts ≥ 10,000/μL (*p*-values: 0.05 and 0.04, respectively). In comparison with seven similar studies, all showed more seizure clusters, partial seizures, and a shorter interval between AGE onset and seizures in afebrile patients than in febrile patients. Contrarily, afebrile patients had longer seizure duration and lower serum hepatic transaminases than febrile patients.

**Conclusion:** Although fever partially influenced the clinical features of AGE-related seizures, febrile CwG might have pathophysiology distinctly different from that of febrile seizures. Comprehensive knowledge in discerning febrile and afebrile CwG can help to avoid unnecessary diagnostics tests, and anticonvulsants use.

## Introduction

Acute gastroenteritis (AGE) is a highly preventable and clinically significant health issue in pediatric medicine. It is the third most common cause of death in children <5-years-old in the developing world (1). It is also well-known that seizures occasionally accompany AGE in pediatric cases (2). When seizures occur concomitantly with AGE, it is usually referred to as benign convulsions associated with mild gastroenteritis (CwG) (3, 4). CwG diagnosis criteria include previously healthy children who present with afebrile (mean temperature <38.0°C), brief, apparently generalized seizures accompanying gastroenteritis, but with no signs of dehydration or electrolyte disturbance (5). Recently, seizures accompanying an episode of acute viral (or presumed viral) AGE have been increasingly recognized in East Asia, particularly in Japan, Taiwan, South Korea, Hong Kong, and recently, from Europe and the United States, where it occurs in ~1–2% of all AGE cases (5–9).

The standard diagnosis criteria of CwG exclude children with fever who are concurrently suffering from AGE-related seizures (10, 11). However, in clinical settings, a fever accompanying an AGE episode is not a rare scenario. High-grade fevers accompanying AGE may induce seizures, particularly among infants and young children, who are more susceptible to febrile stimuli due to immaturity of the brain (12). Distinguishing febrile and afebrile gastroenteritis-related seizures as separate entities could be difficult because too often in a clinical scenario, both the fever and other causes may simultaneously induce the seizure of a child suffering from AGE (13). To date, there is insufficient data that compares the characteristics of febrile and afebrile seizures during a mild AGE episode. Previous studies suggest that the clinical features of febrile seizures related to AGE may be closer to classical CwG than to simple febrile seizures (14–16). However, it is still unclear how great of an impact fever has on AGE-associated seizures, and whether febrile AGE-related seizures can be deemed a new subtype (atypical) of CwG or are just a variant of febrile seizure (17, 18).

In this study, we retrospectively evaluated the clinical and laboratory characteristics and outcomes of children with febrile or afebrile seizures associated with mild AGE. We aimed to assess the impact of fever on AGE-related seizures and to clarify whether AGE-related seizures should be reclassified into two distinct entities.

## Materials and Methods

### Patients

Electronic databases were retrospectively analyzed to identify consecutive children who had been admitted to the pediatric department of Kaohsiung Medical University Hospital due to seizures associated with a mild AGE episode between January 2008 and December 2017. We defined “mild AGE” as a single AGE episode without moderate to severe complications, such as dehydration, electrolyte derangement, or hypoglycemia. Patients with AGE were diagnosed at admission or during hospitalization by the attending pediatrician based on symptoms including vomiting and/or diarrhea. Patients were categorized into the febrile group if they had a central body temperature higher than 38°C within 24 h of AGE-related seizures; otherwise, patients with an average temperature throughout the AGE course were categorized into the afebrile group. This study was approved by the Institutional Review Board of Kaohsiung Medical University Hospital (KMUHIRB-SV(I)-20180019).

The enrollment criteria for subjects included being previously healthy, age <18 years, and having no history of epilepsy. As other abnormal medical conditions are capable of inducing seizures, exclusion criteria included having a history of abnormal neurological development, meningitis, encephalitis, encephalopathy, cerebral trauma, brain tumor, hypoxia, or any other underlying diseases of the central nervous system. Ultimately, 71 children, namely 41 afebrile cases and 30 febrile cases, were enrolled in the study.

### Clinical and Laboratory Characteristics of Children With Afebrile and Febrile Seizures Associated With Age

We retrospectively analyzed patients' background, demographic data, symptoms of AGE, seizure semiology and duration, clustering of seizures (≥2 episodes), the time from the onset of AGE to the first seizure, and family and personal histories of febrile seizures or epilepsy. The general physical and neurologic examinations from the emergency department were collected. Emergency anticonvulsant treatment was documented as were any prescriptions given in the emergency department or pediatric wards.

Laboratory data and clinical symptoms were collected at the time of admission. Data of blood examination included white blood cell (WBC) counts, blood glucose levels, serum electrolytes, hepatic enzymes, and venous blood gas. Tests for viral enteropathogenic antigens (rotavirus, norovirus, and adenovirus) in stool specimens were performed using commercially available immunochromatographic kits. Blood and stool samples were cultured for aerobic pathogens. Lumbar puncture for cerebral spinal fluids (CSF) analysis, electroencephalography (EEG), and brain computed tomography (CT) and magnetic resonance imaging (MRI) and were performed if the attending pediatrician decided they were necessary. EEG reports were obtained within 7 days after the seizures using an international 10–20 recording system. The results of the neuroimaging studies and EEG were reviewed and confirmed radiologists and pediatric neurologists.

### Statistical Analysis

The chi-squared (*x*^2^) or Fischer exact test was used to compare categorical variables according to the analyzed sample size. The Student *t*-test was used to compare continuous variables between the febrile and afebrile groups. For all tests, a 2-sided *p* < 0.05 was considered to indicate statistical significance. We performed all statistical analyses using SPSS software (version 19.0; SPSS, Chicago, IL).

## Results

During the study period, we enrolled 71 infants and young children who presented with seizures during solitary mild AGE. The mean age was 32.2 ± 27.6 months (range, 5–156 months); 31% of subjects were younger than 18 months. The patient population consisted of 39 boys and 32 girls. All enrolled cases were further divided into an afebrile (41 children) or febrile (30 children) CwG group. Table 1 demonstrates the comparisons of the clinical and demographic characteristics and seizure patterns between the afebrile and febrile groups.

**Table 1:** Clinicodemographic and seizure characteristics of children with afebrile and febrile seizures associated with mild acute gastroenteritis.

	**Group**		***P*-value**
	**Afebrile (*n* = 41)**	**Febrile (*n* = 30)**	
Age, mo, mean ± SD (range)	26.8 ± 17.9 (5–76)	32.8 ± 20.9 (11–96)	0.38
Male gender	19 (46.3%)	20 (66.7%)	0.08
Past history of febrile seizures	1 (2.44%)	2 (6.67%)	0.78
Family history of febrile seizure or epilepsy	7 (17.07%)	4 (13.33%)	0.40
**Associated AGE symptoms**			
Vomiting only	8 (19.5%)	7 (23.3%)	0.70
Diarrhea only	12 (29.3%)	8 (26.7%)	0.81
Vomiting and diarrhea	21 (51.2%)	15 (50.0%)	0.52
**Seasonal distribution of seizures**			0.69
September–November	10 (24.39%)	4 (13.33%)	
December–February	11 (26.83%)	10 (33.33%)	
March–May	12 (29.27%)	9 (30.00%)	
June–August	8 (19.51%)	7 (23.33%)	
**Seizure semiology**			
Generalized	18 (43.9 %)	22 (73.3%)	
Partial	17 (41.5 %)	5 (16.7%)	0.04
Atonic	6 (14.6%)	3 (10%)	
Patient with clustered seizures	25 (61.0%)	7 (23.3%)	<0.01
Seizure frequency, episodes, mean ± SD	2.17 ± 1.41	1.50 ± 1.31	0.04
Family history of febrile seizure or epilepsy	7 (17.07%)	4 (13.33%)	0.40
Interval from the onset of gastrointestinal symptoms to the first seizure, d, mean ± SD	1.85 ± 1.31	1.20 ± 1.16	0.03
Seizure duration ≥ 5 min	19 (46.3%)	6 (20.0 %)	0.04

*Values are expressed in number of patients (%) otherwise noted*.

*SD, standard deviation*.

As shown in Table 1, there were more clustered seizures and total seizure frequency in afebrile patients than in febrile patients (both *p* < 0.05). The generalized tonic-clonic seizure was the most prevalent seizure type in both groups. The incidence of partial seizures was higher in the afebrile group than in the febrile group. Moreover, the seizure characteristics among the febrile group and the afebrile group showed significant differences.

**Figure 1:**
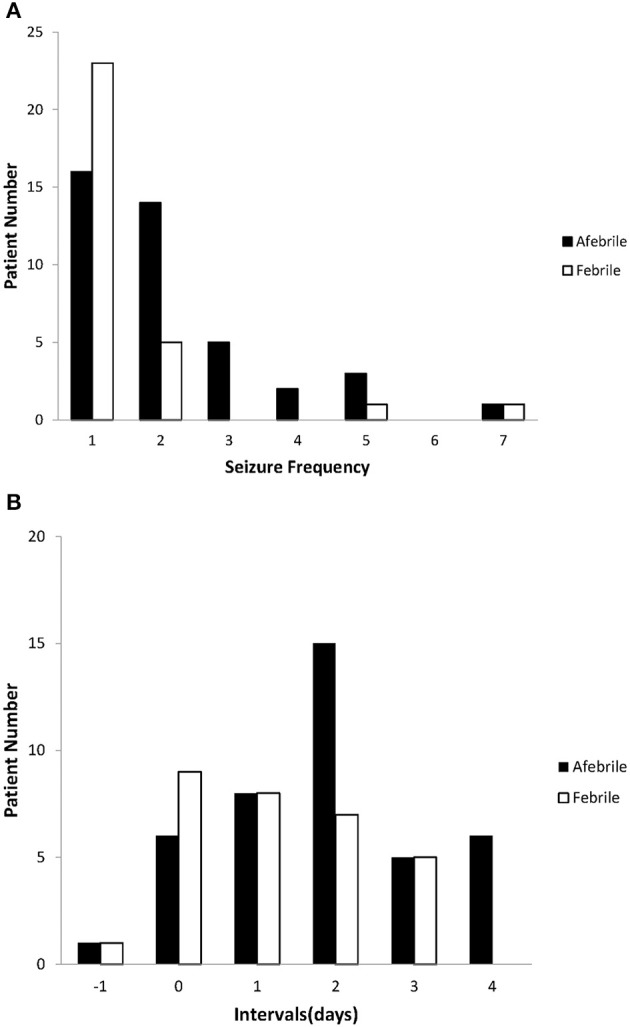
Seizure characteristics of afebrile and febrile groups. **(A)** The seizure frequency of the afebrile and febrile groups. The most common seizure frequency was once in both groups; however, compared to febrile patients, afebrile patients tended to experience seizure clusters (≥ 2 episodes) more frequently. **(B)** The intervals between the onset of AGE symptoms and the first seizures in both the afebrile and febrile groups. Negative numbers indicate that seizures preceded the onset of gastroenteritis. Nine febrile patients and six afebrile patients had seizures on the same day AGE symptoms appeared. One afebrile patient and one febrile patient experienced seizures prior to the onset of AGE symptoms.

Patients of both groups had a seizure frequency of one; however, compare to the febrile patients, the afebrile patients experienced seizure clusters more frequently (Figure 1A).

We observed various intervals between the onset of AGE symptoms and the first seizures of both groups (Figure 1B). There was a shorter mean interval between the onset of AGE symptoms and the first seizures in the febrile group than in the afebrile group (*p* = 0.03). In particular, we found that afebrile patients with a duration of AGE symptoms lasting 2 days or more tended to experience clustered seizures, compared to febrile patients (2.45 ± 1.45 vs. 1.47 ± 1.05, *p* = 0.05, *x*^2^-test).

As shown in Table 2, WBC counts were higher in the febrile group than in the afebrile group (*p* = 0.03). Interestingly, analysis of the subgroups showed a higher incidence of clusters of seizures in the afebrile patients with WBC ≥ 10,000/μL (seizure frequency: 2.51 ± 1.01 vs. 1.22 ± 0.45, *p* = 0.04, *x*^2^-test). Although both groups had mild hyponatremia, lower serum sodium levels and higher hepatic enzymes of aspartate aminotransferase (AST) and alanine transaminase (ALT) levels were observed in the febrile group than the afebrile group (all *p* < 0.05). CSF exams were performed in 4 (13.33%) febrile patients and eight (19.51%) afebrile patients. All 12 patients had normal CSF findings.

**Table 2:** Laboratory test results of children with afebrile and febrile seizures associated with mild acute gastroenteritis.

	**Group**		***P*-value**
	**Afebrile (*n* = 41)**	**Febrile (*n* = 30)**	
**Blood examinations (mean** **±** **SD)**			
WBC (per μL)	9,330.24 ± 3,514.59	11,598.0 ± 5,118.79	0.03
Na^+^ (mEq/L)	135.9 ± 2.8	134.2 ± 3.4	0.02
AST (IU/L)	40.13 ± 11.30	54.37 ± 37.41	0.04
ALT (IU/L)	22.59 ± 9.15	25.25 ± 16.42	<0.01
PH	7.34 ± 0.08	7.34 ± 0.07	0.96
PaCO^2^	37.59 ± 9.46	40.80 ± 15.39	0.39
HCO3^−^	19.61 ± 3.77	20.88 ± 4.81	0.32
**CSF analysis (*****n*** **=** **12)**	8 (19.51%)	4 (13.33%)	0.71
Normal results	8 (100%)	4 (100%)	
**Arranged enteropathogen surveys (*****n*** **=** **50)**	22 (53.7%)	24 (80%)	
Rotavirus	3 (13.6%)	12 (50%)	0.02
Norovirus	6 (27.3%)	4 (16.7%)	0.61
Adenovirus	3 (13.6%)	2 (8.3%)	0.92
Salmonella type C		2 (8.3%)	
Unidentified pathogens	10 (45.5%)	4 (16.7%)	0.07

*Values are expressed in number of patients (%) otherwise noted*.

*SD, standard deviation; WBC, white blood cell; AST, aspartate aminotransferase; ALT, alanine transaminase; CSF, cerebrospinal fluid*.

Forty-six patients, namely 22 afebrile patients and 24 febrile patients, were examined for causative enteropathogens (Table 2). The most commonly identified enteropathogens were rotavirus, which was detected in the stool of 15 cases (32.6%), followed by norovirus in 10 cases (21.7%), and adenovirus in five cases (10.9%). Two febrile cases were infected with Salmonella group C, which was isolated in stool and blood cultures. Compared to the non-rotavirus infected patient group (*n* = 35), fever and male gender were more frequently observed in the rotavirus infected group (*p* = 0.04 and *p* = 0.05, respectively; data not shown). The meantime from gastrointestinal symptom onset to first seizure, duration of seizure, presence of cluster seizures, and mean seizure frequency was not significantly different between the rotavirus and non-rotavirus groups.

Table 3 shows the comparison of EEG, neuroimaging, treatment, and outcome between groups. In the febrile group, slow background activity was reported in one patient, and paroxysmal discharge was observed in another patient. In the afebrile group, spikes in the asymmetric central region (*n* = 6) were most frequently observed, followed by generalized background slow-waves (*n* = 2) and slow-wave bursts (*n* = 1). Of note, abnormal EEG results were only observed in the non-rotavirus infected group (5/20, 25%). Ultimately, follow-up EEGs taken 2–6 months later in 42 patients were normal. Normal neuroimaging findings were reported in all but one afebrile patient whose brain MRI showed a transient splenic lesion of the corpus callosum.

**Table 3:** Data of electroencephalography, neuroimaging, treatment and outcome of afebrile and febrile groups.

	**Group**		***P*-value**
	**Afebrile (*n* = 41)**	**Febrile (*n* = 30)**	
**Interictal EEG examination**	27 (65.9%)	19 (63.3%)	0.97
Normal	16 (59.3%)	17 (89.5%)	
Abnormal	11 (40.7%)	2 (10.5%)	0.04
**Neuroimaging studies**	12 (29.3%)	4 (13.3%)	0.19
Brain CT	10 (24.4%)	3 (10%)	
Brain MRI	4 (9.8%)^*^	1 (3.3%)	
Antiepileptic drugs use	15 (36.59%)	8 (26.67%)	0.38
**Hospitalization and PICU admission**			
Overall admission rate	29 (70.7%)	25 (83.3%)	0.34
Length of hospitalization, d, mean ± SD	4.59 ± 1.57	5.96 ± 2.81	0.04
PICU admission	5 (12.2%)	1 (3.3%)	0.37
PICU stay, d, mean ± SD	4.2 ± 1.92	3	
**Prognosis and outcomes**			
Recurrence AGE-associated seizures	5 (12.2%)	6 (20%)	0.57
Development of epilepsy	6 (37, 16.22%)	3 (30, 10%)	0.69

*Values are expressed in number of patients (%) otherwise noted*.

* *Including two patients also received brain CT examinations*.

*SD, standard deviation; EEG, electroencephalography; CT, computed tomography; MRI, magnetic resonance imaging; PICU, pediatric intensive care unit*.

Emergency anticonvulsant treatment were administrated in a single dose of an intravenous benzodiazepine, including diazepam, lorazepam, or midazolam, as the first-line agents for children who had repeated seizures after admission. Nineteen patients responded well to the initial benzodiazepine treatment; however, four patients were unresponsive to the maximum of three repeated doses. Three patients were given intravenous levetiracetam, and one patient was given phenytoin as a secondary anticonvulsant agent. These medications were effective in all four patients. Fifty-four patients (76.06%) required subsequent hospitalization. Five afebrile patients and one febrile patient required further PICU admission, mainly due to seizure clusters (50%). Nevertheless, all hospitalized patients from both groups were discharged uneventfully, without mortality or severe neurological sequelae.

During the follow-up period, which ranged from 12 to 36 months (mean duration: 14.5 months), five afebrile patients and six febrile patients experienced recurrences of seizures during a new gastroenteritis episode. Three patients (4.2%) suffered from uncomplicated febrile seizures associated with other infections. The recurrence of seizure attacks was not statistically significant between the two groups. Finally, no patient was diagnosed with epilepsy during the follow-up visit at the outpatient clinic. EEGs taken 4–12 months after seizure onset showed unremarkable in 12 patients. Psychomotor development was within the normal limits in all children at the latest follow-up.

## Discussion

Afebrile seizures associated with AGE have been categorized independently as CwG, which was first proposed in 1982 (3). This benign seizure type typically occurs in 6 months to 3-years-old children and has a tendency to occur in a repetitive or clustered manner (11). The presence of seizures during AGE may be attributed to multiple factors, including fever, dehydration, ad electrolyte imbalance, or maybe related explicitly to some pathogens, such as shigella and campylobacter (19–21). To date, the majority of CwG studies excluded febrile cases even though febrile seizures sometimes occur during an AGE episode (5, 6). There is still a controversy regarding whether febrile seizures themselves during AGE should be placed in the category of CwG (16–18). On the other hand, febrile seizures, with a peak incidence between 12 and 18 months of age, likely overlap the high susceptible age group of CwG (22, 23). In this study, we found that the afebrile and febrile patients with AGE-related seizure shared similar clinical and laboratory features; however, there were still some distinct characteristics between the two groups. These findings imply that the seizures in the febrile group more closely resembled CwG than febrile seizures in general. Importantly, ultimately favorable outcome was obtained in both groups. We suggested that pediatric febrile AGE-related seizures may be considered a subgroup of atypical CwG rather than purely febrile seizures induced by AGE.

To our knowledge, seven comparative studies have analyzed the differences of clinicolaboratory features between afebrile and febrile CwG caused by various enteropathogens, which are mainly attributed to rotavirus and norovirus infections (14–18, 24, 25). As shown in Table 4, our data, consistent with the majority of previous studies, showed that afebrile patients (typical CwG) had more clusters of seizures, greater total seizure numbers, more partial seizures, and shorter intervals between AGE symptom onset and first seizure than febrile patients (atypical CwG). While febrile seizures had a male gender predominance, females seemed to be more susceptible to typical (afebrile) CwG, as demonstrated in our study and the previous studies (7, 15, 24). Compared to the febrile patients, the afebrile patients also had a higher rate of abnormal EEGs and lower serum sodium levels. However, we observe several findings contrary to the findings from previous studies. Our afebrile patients tended to have longer seizure durations (≥5 min), in contrast to the result of Lee et al. (17). There were no differences in the family or personal histories of febrile seizures between our febrile and afebrile groups (15, 17, 18). There are higher serum hepatic transaminase levels in our febrile patients. Noteworthily, we found that clusters of seizures occurred more frequently in afebrile patients with a duration of AGE symptoms lasting 2 days or more or leukocytosis (WBC ≥ 10,000/μL). Except for the age (7), these findings were not reported as an association with seizure frequency in afebrile CwG. In brief, our findings implicate that perhaps in the context of AGE-related seizures, the absence of fever may hold particular significance for the clinical and laboratory features distinct from the patients with fever (6).

**Table 4:** Comparison of clinicolaboratory features among studies of afebrile and febrile seizures associated with acute gastroenteritis.

	**Lloyd et al. (25)**	**Zifman et al. (16)**	**Lee and Chung (17)**	**Kang et al. (15)**	**Ueda et al. (14)**	**Hu et al. (24)**	**Higuchi et al. (18)**	**Present study**
Case number	34	44	59	59	14	108	126	71
(aF:F)	(23:11)	(18:26)	(32:27)	(42:17)	(8:6)	(59:49)	(76:50)	(41:30)
Study design	Retrospective	Retrospective	Retrospective	Retrospective	Retrospective	Retrospective	Prospective	Retrospective
Gender (Female predominant)	NS	NA	NS	aF>F	F>aF	aF>F	NS	aF>F
Total number of seizures	NA	NA	aF>F	aF>F	NS	NA	NA	aF>F
Presence of clustered seizures	NA	NA	aF>F	aF>F	NS	aF>F	aF>F	aF>F
Partial seizure predominant	NS	NA	aF>F	aF>F	NS	NA	NS	aF>F
Prolonged seizure (≥5 min)	NS	NS	F>aF	NS	NS	NA	NS	aF>F
Personal history of febrile seizures	NA	NS	F>aF	F>aF	NA	NA	F>aF	NS
Family history of febrile seizures or epilepsy	NA	NA	NS	NS	NS	NA	F>aF	NS
Interval between AGE onset and first seizures	NA	NA	aF>F	aF>F	aF>F	aF>F	aF>F	aF>F
Pathogen Identification	Exclusively rotavirus	Rotavirus only in 2 patients	Rotavirus and Salmonella B	Exclusively rotavirus	Rotavirus and norovirus	Exclusively norovirus	Mixed rotavirus, norovirus, and adenovirus	Rotavirus, norovirus, adenovirus, and Salmonella C
EEG abnormalities	NS	NS	aF>F	NS^*^	NS	NA	NS	aF>F
Neuroimaging arrangement	aF>F	NA	NA	aF>F	NS	NA	aF>F	NS
WBC	NA	NA	NA	F>aF	NS	F>aF	NA	F>aF
Serum sodium level	NA	aF>F	NS	aF>F	NS	NS	aF>F	aF>F
Elevated AST	NA	NA	NA	aF>F	NS	aF>F	aF>F	F>aF
Elevated AST	NA	NA	NA	NS	NS	NA	aF>F	F>aF

* *Abnormalities were only observed in the afebrile group*.

*^†^Mild hyponatremia were noted in both groups*.

*aF, afebrile group; F, febrile group; NA, not available; NS: not significant; AGE, acute gastroenteritis; EEG, electroencephalography; WBC, white blood cell; AST, aspartate aminotransferase; ALT, alanine transaminase*.

It is known that fever can influence cellular processes, such as the electrical activity of neurons, resulting in a hyperexcitable brain in young children (13). However, few studies had discussed the impact of fever on clinicolaboratory features among children with AGE-related seizures (26, 27). We observed several distinct findings between the afebrile and febrile groups. First, both groups had mild hyponatremia; however, serum sodium levels were even lower in the febrile group than in the afebrile group (15, 16, 18). Zifman et al. reported hyponatremia in children with AGE-related seizures, particularly in febrile patients (16). It has been postulated that hyponatremia lowers the seizure threshold in children with recurrent febrile convulsions (28, 29). Whether mild hyponatremia can further lower the seizure threshold in children with AGE, irrespective of body temperature, requires a future investigation. Second, in our study, higher hepatic transaminases were found in the febrile patients than in those who were afebrile, which is contrary to some previous studies (15, 18, 24). Although rotavirus-associated AGE is known to be complicated by elevated hepatic transaminase levels (30), we did not observe such a correlation. AST is abundantly expressed in several non-hepatic tissues, whereas ALT is found at low concentrations in tissues other than the liver. We postulate that elevated hepatic transaminase levels may represent not only hepatic injury but also non-specific tissue damage caused by inflammation, fever, or by an extra-intestinal effect. Lastly, we found fever accompanying more frequently in patients with rotavirus infection, consistent with previous comparative studies of febrile and afebrile CwG groups (14, 18). This finding implies that rotavirus might play a more significant role in febrile CwG than in afebrile CwG. Thus, in the year that the rotavirus vaccine was introduced in Asian countries, rotavirus CwG was expected to decrease, but the incidence of atypical rotavirus CwG associated with fever might increase due to uncovered rotavirus serotypes (31).

Previous studies showed that the majority of children with CwG had normal interictal EEGs, albeit few cases might have temporary abnormalities like slow background activity and sharp wave discharge (11, 32). Interestingly, our study showed that the afebrile patients had more temporary EEG abnormalities, in particular asymmetrically focal spikes, compared to the febrile patients. This finding may be explained by the higher rate of seizure clusters and partial seizures, and the longer seizure duration in the afebrile patients (33). We also found that abnormal EEG results were only found in the non-rotavirus infected group. We are still unclear about the association between enteropathogens and EEG abnormalities, particularly in afebrile CwG cases. This correlation may require a more extensive multicenter study with more confirmatory identifications of enteropathogens. Nevertheless, although interictal abnormalities were initially present on EEG, these reverted to normal during the follow-up period, consistent with the relatively benign nature of AGE-related seizures (9).

Regardless of fever, our patients generally have a favorable prognosis as previous studies (9, 34). More extended stay of hospitalization in our febrile patients than afebrile patients may be related to the waiting time for fever subside before the ensured discharge, rather more complications caused by fever. The probability of CwG recurring, suffering from a febrile seizure, or developing epilepsy appeared to be low. The seizure recurrence rate during new episodes of AGE was 12.6% in our study, and 3–20% in the previous case series studies (10, 35). Notably, 4.2% of our patients subsequently suffered from febrile seizures associated with infection other than AGE, which is consistent with the previously estimated rate of 2.5–6.7% (9).

This study has several limitations. The main limitation is related to the retrospective design of this study. To our knowledge, the majority of previous studies regarding either afebrile or febrile CwG are designed retrospectively (9, 36). Nevertheless, our findings may provide informative variables for future studies with a prospective design. Second, due to the retrospective nature of the study, not all the patients received a full work-up with causative enteropathogen, and a proportion of the subjects in both groups had negative results for an enteropathogen. Interestingly, a recent study in southern Taiwan reported an unidentified pathogen, which accounted for most of the pediatric AGE infection that required hospitalization. Other, less prevalent, enteropathogens causing AGE included salmonella, norovirus, and rotavirus (37). Lastly, there was a possibility of enrolling patients with febrile seizures in the febrile CwG group. However, we did not find that the personal or family histories for febrile seizures were significantly more prevalent in our febrile group than in the afebrile group. Careful interpretation is required because of the nature of the study's design and lack of long-term follow-up data.

## Conclusion

Our findings, as well as the reviewed evidence from the comparative studies, indicate that children with febrile AGE-related seizures may be categorized as atypical CwG. Atypical CwG may have a pathophysiology distinct from that of febrile seizures. Further, we suggested that afebrile patients tended to experience clustered seizures if they had an extended AGE duration lasting 2 days or more and leukocytosis. Knowledge of the characteristics of both febrile and afebrile CwG is essential for pediatricians to avoid unnecessary hospitalizations, invasive procedure, neuroradiological examinations, and above all, long-term anticonvulsant treatments, which have potential side effects on children.

## Data Availability Statement

The datasets used and/or analyzed during the current study are available from the corresponding author on reasonable request.

## Ethics Statement

The studies involving human participants were reviewed and approved by Institutional Review Board of Kaohsiung Medical University Hospital. Written informed consent of participants was waived with an approval by the Institutional Review Board.

## Author Contributions

Y-ZW contributed to conception and design, acquisition of data, or analysis and interpretation of data, and drafting the manuscript for intellectual content. Y-HL contributed to acquisition and interpretation of data. C-MT contributed to acquisition of data. Y-HT contributed to acquisition of data. T-HC contributed to conception and design, acquisition of data, revising the manuscript critically for important intellectual content, and final approval of the version to be published. All authors read and approved the final manuscript.

### Conflict of Interest

The authors declare that the research was conducted in the absence of any commercial or financial relationships that could be construed as a potential conflict of interest.
